# A Sign to Heaven: aVR Lead Elevation and Myocardial Infarction

**DOI:** 10.1100/tsw.2011.63

**Published:** 2011-03-22

**Authors:** Amir M. Nia, Natig Gassanov, Hannes Reuter, Fikret Er

**Affiliations:** Department of Internal Medicine III, University of Cologne, Cologne, Germany

**Keywords:** aVR lead elevation, ST-segment elevation in aVR, left main coronary artery obstruction, LMCA obstruction, proximal LAD stenosis

## Abstract

Isolated ST-segment elevation only in the aVR lead, reflecting an acute myocardial infarction due to a left main coronary artery occlusion, was ignored as part of physicians' training in emergency medicine for a long time. The recognition of aVR lead elevation is becoming more accepted as a mandatory diagnostic tool, in particular for physicians working at emergency departments. We report a typical myocardial infarction with total occlusion of the proximal part of the left anterior coronary artery, presenting with ST-segment elevation in the aVR lead, which was misinterpreted as diffuse ischemia. The lacking mandatory awareness of this entity endangered prompt and correct treatment.

## CASE

A 63-year-old man was referred from a tertiary hospital's emergency triage to our cardiology department with the diagnosis of acute coronary syndrome with global ST-segment depressions. On admission, his blood pressure was 100/60 mmHg with a heart rate of 90 beats per minute. He complained of moderate chest pain of several days duration, but 1 h before admission, he noticed an increasingly severe thoracic discomfort, which caused him to seek medical attention. In his history, Parkinsonism, arterial hypertension, and hyperlipidemia were known. Prior to the last days, he had never felt angina pectoris. The initial electrocardiogram (ECG) was in his hands (Fig. 1). At first, distinct ST-segment depressions in eight of the 12 ECG leads were apparent. Nevertheless, the most striking ECG abnormality was characterized by a prominent ST-segment elevation (STE) of almost 5 mm (0.5 mV) in the aVR lead, highly suggestive of a left main coronary artery (LMCA) stenosis[1,2,3,4]. Immediate cardiac catheterization was done with the presumed diagnosis of an acute STE myocardial infarction (STEMI). During catheterization, the patient decompensated based on recurrent ventricular fibrillation. We started cardiopulmonary resuscitation and performed cardiac catheterization under resuscitation. Indeed, the coronary angiogram revealed a complete occlusion of the LMCA (Fig. 2; Panel A). The initial stable situation was probably ascribable to the prominent and intact large right coronary artery (not shown). After approximately 10 min, a return of spontaneous circulation could be achieved. Due to the worsening of the hemodynamic status, a rescue angioplasty was done for bridging to the coronary artery bypass grafting (Fig. 2; Panel B). The patient underwent successful surgical intervention and could be discharged as cardiopulmonary stable.

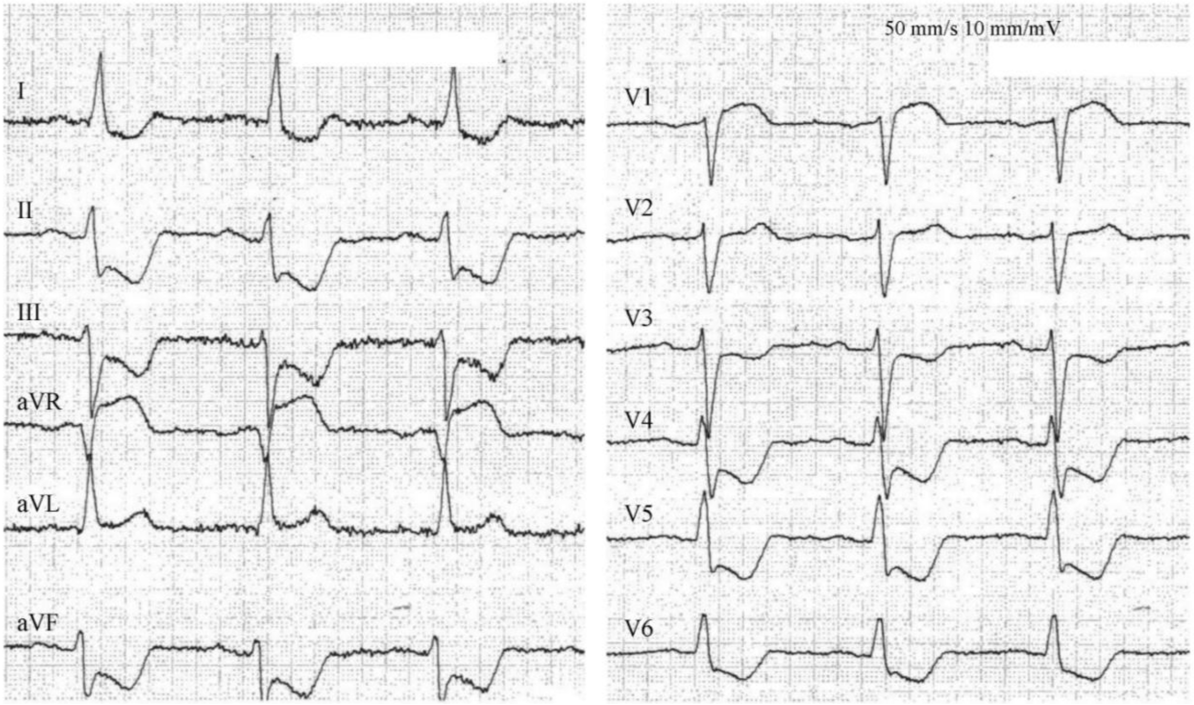

**FIGURE 1.** 12-Lead ECG reveals a prominent ST-segment elevation in the aVR lead.

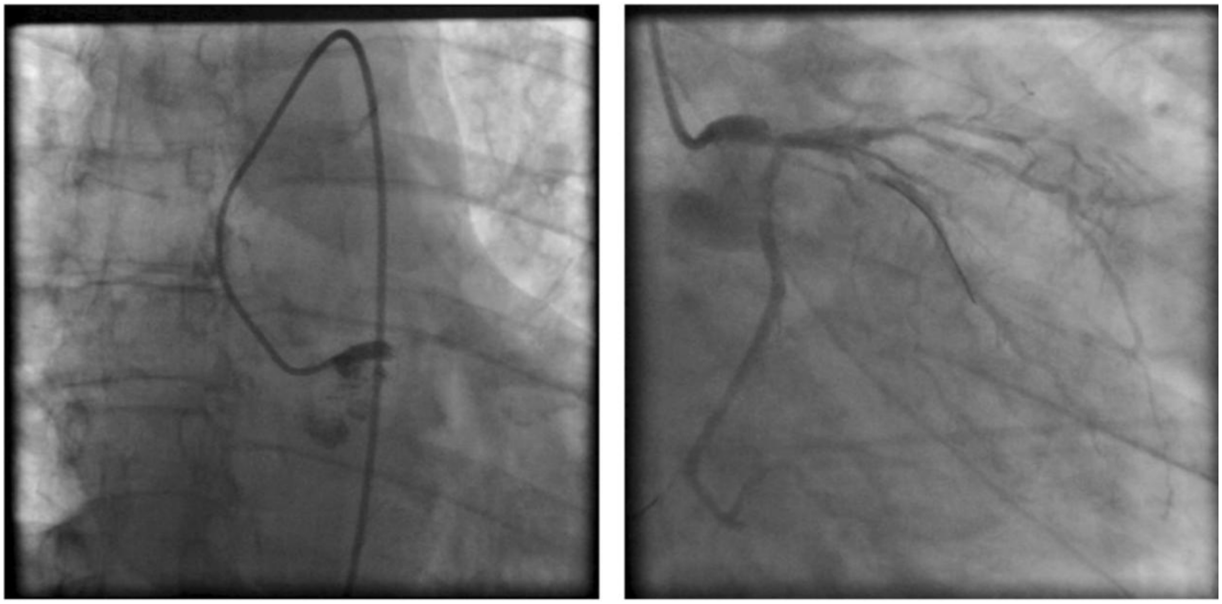

**A** **B**

**FIGURE 2.** Coronary angiograms. (A) Anterior-posterior view displays a complete occlusion of the LMCA. (B) Right anterior oblique 30°/caudal 20° view displays a marginal reperfusion after rescue angioplasty of the LMCA.

The 12-lead ECG is a widely available bedside test, especially in the emergency department, for urgent triage. Although each lead provides specific information, the aVR lead has been frequently overlooked in the past[2,5,6]. A review of the literature uncovers that aVR lead changes are widely ignored[5,7,8,9]. A very interesting study was performed to investigate ECG interpreters' disregard of the aVR lead[5]. An experienced medical staff was asked to interpret complex ECGs, but the aVR lead had been replaced by the –aVR lead (reversed aVR lead with putative positive vector) on all of these recordings. The vast majority of interpreters (80–94%) did not detect when the aVR lead had been reversed[5]. Probably, the usually negative QRS vector of the aVR lead may lead to its underestimation. However, the tracing in this lead can be used to obtain a unique view directly into the right ventricular outflow tract and the basal portion of the interventricular septum[6]. Thus, it is important to use the aVR lead as an essential part of the ECG interpretation[2,3,10]. There are further aVR lead findings worthy of discussion, such as PR-segment elevation indicating acute pericarditis or prominent R' waves indicating tricyclic antidepressant poisoning[10,11]. Moreover, several levels of evidence substantiate that STE in the aVR lead is highly associated with the left main, the left anterior descending coronary artery (LAD), and 3-vessel coronary artery disease[1,2,3,4,12]. Yamaji et al. reported that STE in the aVR lead greater than that in lead $V_1$ may be useful for predicting acute LMCA obstruction, which is a rare angiographic finding and requires immediate intensive treatment[3]. During the last years, this finding could be confirmed by other colleagues[2,4,12]. The most likely explanation of less STE in lead $V_1$ in patients with LMCA disease compared to those with LAD disease may be the result of additive posterior wall ischemia in LMCA-diseased patients[3]. Contrary to patients with LAD disease, LMCA obstruction induces posterior wall ischemia through disturbance of left circumflex artery blood flow[3]. Due to this, the posterior wall–induced electrical force counterbalances the ischemia-induced electrical force in the anterior wall, leading to more prominent STE in the aVR lead than in the chest leads[2,3].

As exemplified in our case, STE in the aVR lead is often misinterpreted and typically neglected. Apparently, this case report displays that the general criteria for STEMI might be incomplete and that an isolated STE in the aVR lead reflects a STEMI due to occlusion of the LMCA[2,3,4]. We think that it is mandatory that this "Sign to Heaven" be known and recognized by all physicians interpreting ECGs in their daily clinical work.
